# Effects of the combination of TRC105 and bevacizumab on endothelial cell biology

**DOI:** 10.1007/s10637-014-0129-y

**Published:** 2014-07-05

**Authors:** Yingmiao Liu, Hongyu Tian, Gerard C. Blobe, Charles P. Theuer, Herbert I. Hurwitz, Andrew B. Nixon

**Affiliations:** 1 Department of Medicine, Duke University Medical Center, Box 2631, Durham, NC 27710 USA; 2 Tracon Pharmaceuticals, Inc., San Diego, CA 92121 USA

**Keywords:** TRC105, Endoglin, Bevacizumab, Angiogenesis, Endothelial cells

## Abstract

Endoglin, or CD105, is a cell membrane glycoprotein that is overexpressed on proliferating endothelial cells (EC), including those found in malignancies and choroidal neovascularization. Endoglin mediates the transition from quiescent endothelium, characterized by the relatively dominant state of Smad 2/3 phosphorylation, to active angiogenesis by preferentially phosphorylating Smad 1/5/8. The monoclonal antibody TRC105 binds endoglin with high avidity and is currently being tested in phase 1b and phase 2 clinical trials. In this report, we evaluated the effects of TRC105 on primary human umbilical vascular endothelial cells (HUVEC) as a single agent and in combination with bevacizumab. As single agents, both TRC105 and bevacizumab efficiently blocked HUVEC tube formation, and the combination of both agents achieved even greater levels of inhibition. We further assessed the effects of each drug on various aspects of HUVEC function. While bevacizumab was observed to inhibit HUVEC viability in nutrient-limited medium, TRC105 had little effect on HUVEC viability, either alone or in combination with bevacizumab. Additionally, both drugs inhibited HUVEC migration and induced apoptosis. At the molecular level, TRC105 treatment of HUVEC lead to decreased Smad 1/5/8 phosphorylation in response to BMP-9, a primary ligand for endoglin. Together, these results indicate that TRC105 acts as an effective anti-angiogenic agent alone and in combination with bevacizumab.

## Introduction

Anti-angiogenic therapies have emerged as prominent approaches for cancer treatment over the past decade [1, 2]. Tumor progression is heavily dependent on angiogenesis for primary tumor growth and metastasis. Anti-angiogenic agents inhibit an organism’s potential to develop new blood vessels and prevent tumor growth by blocking access to oxygen and nutrients. Bevacizumab (Avastin™), the first approved anti-angiogenic drug, binds vascular endothelial growth factor (VEGF) and was approved by Food and Drug Administration for the treatment of metastatic colorectal cancer in 2004 [3]. Bevacizumab is currently approved for multiple cancer indications, based on the prolongation of patient survival, clinically confirming the value of anti-angiogenic therapeutics which target the VEGF pathway [4].

Despite the widespread use of anti-angiogenic agents, the clinical benefit is limited and transient [5]. Such therapies appear to benefit a subset of cancer patients; and those who respond ultimately progress. This phenomenon is not surprising given that angiogenesis is regulated through a complex interplay of multiple pathways. When VEGF-mediated signaling is blocked by bevacizumab, other angiogenic pathways are activated, resulting in drug resistance. Therefore, combining drugs that target different angiogenic pathways may be a more effective strategy. Currently, more than forty anti-angiogenic drugs are being tested in clinical trials [6].

Endoglin is a homodimeric transmembrane glycoprotein highly expressed on proliferating endothelial cells [7, 8]. As a co-receptor for TGF-β and for bone morphogenic protein (BMP), endoglin associates with ALK1, an endothelial cell-specific type-I receptor, to promote downstream Smad 1/5/8 phosphorylation and endothelial cell proliferation, primarily in response to BMP [9]. Recent data by Nolan-Stevaux et al. strongly supports that endoglin-dependent BMP signaling is the critical pathway for Smad 1/5/8 activation in primary HUVEC cells [10]. In contrast, in the absence of endoglin, another type-I receptor, ALK5, promotes downstream Smad 2/3 phosphorylation that maintains a state of EC quiescence. This balance between Smad 1/5/8 and Smad 2/3 phosphorylation regulates EC homeostasis [11]. When Smad 1/5/8 signaling predominates, EC undergo proliferation, migration, and promote angiogenesis; when Smad 2/3 signaling predominates, EC remain quiescent. Consistent with its angiogenic role, endoglin is markedly upregulated on the endothelium of malignancies [8]. Dense staining of endoglin has been observed in the angiogenic blood vessels of more than 10 types of tumor tissues and correlated with poor prognosis [12, 13], suggesting its potential as a target for clinical intervention [14].

TRC105 is a monoclonal antibody that binds endoglin with high avidity and is currently being evaluated in phase 1b and phase 2 clinical trials [15]. TRC105 exhibited promising safety and activity in the first-in-human, phase 1 trial [16]. The phase 1, dose escalation study determined the recommended dose for phase 2 to be 10 mg/kg weekly, or 15 mg/kg every two weeks. Both doses resulted in high circulating TRC105 levels in patients plasma, with peak concentrations ranging from 200 to 600 μg/ml [16].

Due to the fact that TRC105 targets an essential angiogenic pathway distinct from the VEGF pathway targeted by bevacizumab, the combination of both drugs may provide greater activity. In this study, we tested the effects of TRC105 and bevacizumab as single agents, as well as in combination, on EC tube formation, viability, migration, and apoptosis. Further, we assessed the effects of TRC105 on patterns of Smad phosphorylation in HUVEC cells.

## Materials and methods

### Cell culture

Low passage HUVEC cells were purchased from Clonetics/Lonza (Walkersville, MD). HUVEC were cultured in either regular medium containing EBM-2 basic medium supplemented with EGM-2 MV single aliquots; or nutrient-limited medium containing EBM-2 basic medium supplemented with 0.5 % FBS and 30 ng/ml VEGF (Lonza, Walkersville, MD). All cells were maintained in a 37 °C, 5 % CO_2_ incubator. TRC105 (5 mg/ml) was provided by Tracon Pharmaceuticals, Inc. (San Diego, CA). Bevacizumab (25 mg/ml) was from Genentech Inc. (San Francisco, CA).

### HUVEC tube formation

HUVEC were pre-treated with 100 μg/ml TRC105, 100 ng/ml bevacizumab, or both drugs for 8 h in regular medium. Human IgG (Jackson Immuno Research, West Grove, PA) was used as an isotype control. The cells were harvested and maintained in drug containing medium, and 1.5 × 10^4^ HUVEC were inoculated onto pre-polymerized ECMatrix gel (In vitro angiogenesis assay kit, Chemicon, Temecula, CA). After 16 h incubation, cells were visualized using the Axiobserver in the Duke Light Microscopy Core facility (LMCF). Closed polygons were counted, and total tube length measured with the MetaMorph software (MDS Analytical Technologies, Sunnyvale, CA).

### HUVEC viability (MTS assay)

HUVEC were inoculated at 5,000 cell/well onto a 96-well plate. After overnight incubation, cells were treated with either regular or limited medium containing either TRC105, bevacizumab, the combination of both drugs, or IgG control for 72 h with daily medium change. At the termination of the assay, 20 μl MTS tetrazolium compound (CellTiter 96 Aqueous One Solution Cell Proliferation Assay, Promega, Madison, WI) was added to each well, absorbance at 490 nm was recorded 4 h later following the manufacturer’s protocol.

### HUVEC migration

HUVEC (8 × 10^5^/well) were inoculated onto a 6-well plate. After a confluent monolayer had formed overnight, a scratch was introduced with a sterile 200 μl tip. Cell debris was removed by washing with PBS, fresh regular medium containing either IgG isotype control (100 μg/ml), TRC105 (100 μg/ml), bevacizumab (100 ng/ml), or the combination of TRC105 (100 μg/ml) and bevacizumab (100 ng/ml) was supplied. Scratch filling was monitored using a live cell station in Duke LMCF over a period of 16 h. Percentage of scratch filling was calculated as (Distance between gap edges at time point 0 - distance at time point X)/distance at time point 0 *100 %, using MetaMorph software.

### HUVEC apoptosis

HUVEC (1 × 10^5^/well) were inoculated onto a 6-well plate in which a gelatin-coated glass slide had been placed at the bottom. HUVEC were maintained in regular or limited medium with TRC105, bevacizumab, or the combination of both for 72 h. Fresh medium with drugs were applied daily. Cells were then fixed in methanol: acetic acid (3:1) for 5 min at 4 °C, washed three times with PBS, and stained with Hoechst 33,342 (5 μg/ml, Calbiochem, La Jolla, CA) for 10 min at room temperature. Then cells were washed three more times, the slides removed from plate wells, and mounted onto a glass carrier with Vectashield mounting medium for fluorescence (Vector Laboratories, Inc., Burlingame, CA). Apoptotic nuclei were visualized under a fluorescence microscope in Duke LMCF. Ten representative fields were imaged, cells counted, and the ratio of apoptotic nuclei vs. total nuclei was calculated with MetaMorph software.

### Smad signaling in HUVEC

HUVEC (5 × 10^5^/well) were inoculated onto a 6-well plate and incubated overnight. Cells were serum starved in EBM-2, 0.1 % BSA, and 10 mM Hepes for 4 h. Cells were pretreated with TRC105 or isotype control for 1 h, and stimulated with 0.2 ng/ml BMP-9, or 0.25 ng/ml TGF-β1 (R&D systems, Minneapolis, MN) for 1 h. Then cells were put on ice immediately, cell lysate harvested in lysis buffer: 20 mM Hepes, 2 mM MgCl_2_, 1 mM EDTA and EGTA, 150 mM NaCl, 1 % Triton X-100, 0.1 % SDS, and protease inhibitors. After centrifugation at 16,000 × g for 10 min, cell debris and nuclei were removed, and cell lysates were snap frozen before stored at −80 °C freezer.

### Western immunoblots

Cell lysates (10 μg) were separated on a 4–20 % SDS-PAGE gel. Blots were incubated with rabbit-anti-phos-Smad1/5/8, anti-Smad 1, anti-Phos-Smad2, or anti-Smad 2/3 (Cell Signaling, Danvers, MA), as well as mouse anti-β-actin for loading control, overnight at 4 °C. LI-COR specific goat anti-rabbit, or goat anti-mouse IgG secondary antibody (1:5,000) was added and incubated for 1 h at room temperature. Immunoblots were analyzed using the Odyssey imaging system (LI-COR Biotechnology, Lincoln, NE).

## Results

### TRC105 and bevacizumab inhibit HUVEC tube formation

**Fig. 1:**
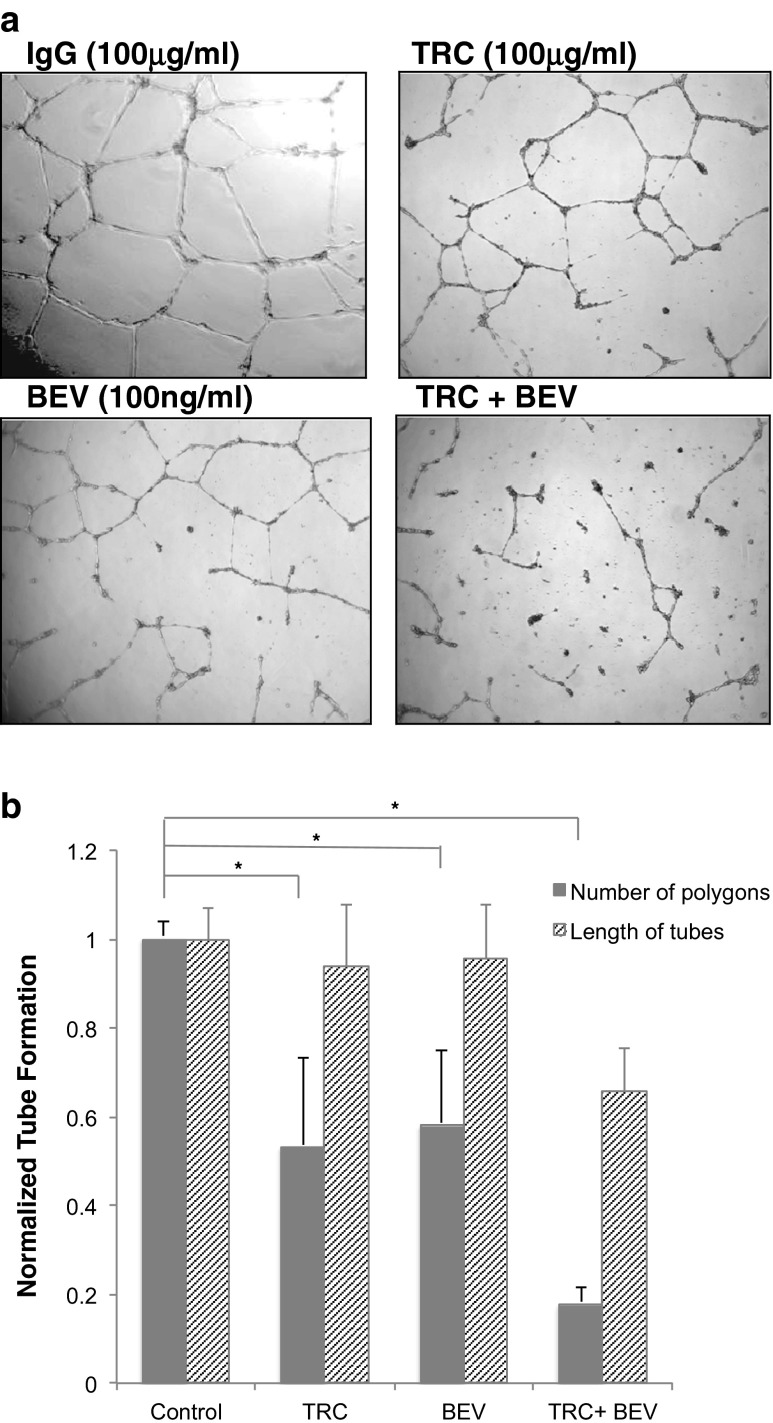
TRC105 and bevacizumab inhibit HUVEC tubular network formation in in vitro angiogenesis assays. **a**. HUVEC cells growing on matrigel overnight in the presence of IgG control (100 μg/ml) formed extensive tube networks. TRC105 (100 μg/ml), bevacizumab (100 ng/ml), or the combination of both drugs was added and the effects on polygon formation and tube length were visualized. **b**. Normalized bar graph showing the fold inhibition on polygon formation and tube length. Each column represents the mean ± SD of three independent experiments. *p < 0.05 compared to IgG control

To evaluate the anti-angiogenic function of TRC105, HUVEC tube formation was first tested. Since TRC105 is a chimeric antibody cloned into an IgG1 backbone, human IgG molecule was used as an isotype control. As shown in Fig. 1a, overnight incubation of HUVEC with IgG control (100 μg/ml) on a matrigel leads to the development of extensive tubular networks, which can be quantified by counting the number of closed polygons and total length of capillary tubes. TRC105 (100 μg/ml) and bevacizumab (100 ng/ml) treated cells showed impaired tube formation, exhibited by less dense tube network and some disrupted regions. Strikingly, when cells were treated with both agents, only partially formed tubes and fewer closed polygons could be detected. After data normalization, polygon formation was inhibited by 40–50 % using either drug alone and by more than 80 % using the combination of both drugs. The combination of drugs is significantly more effective in inhibiting polygon formation than either drug used alone (*p* ≤ 0.05; Fig. 1b). The total length of tubes, an earlier biologic step reflecting mainly cell migration and alignment, was less affected, exhibiting 5–10 % inhibition from each drug alone, and more than 30 % inhibition using the combination of drugs. Overall, these results indicate that both TRC105 and bevacizumab inhibit EC tube formation, and that the combination of drugs achieved more robust inhibition than either drug alone.

### Effect of TRC105 and bevacizumab on HUVEC viability

**Fig. 2:**
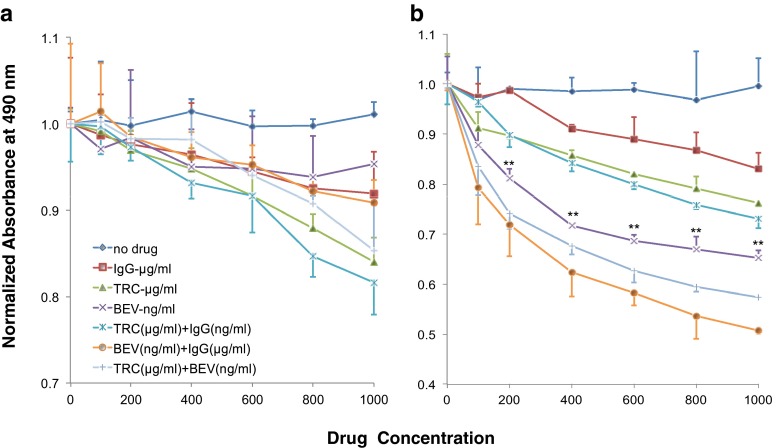
Effect of TRC105 and bevacizumab on HUVEC viability. Subconfluent HUVEC were subjected to TRC105 (100–1,000 μg/ml), bevacizumab (100–1,000 ng/ml), both drugs, or IgG (100–1,000 μg/ml) treatment for 3 days in regular medium (**a**) and limited medium (**b**). Cell viability was measured by MTS tetrazolium assay, and absorbance at 490 nm was reported. Data represent the mean ± SD of three independent experiments. ** *p* < 0.01 compared to IgG control

Since EC tube formation is a comprehensive assay reflecting multiple cellular processes such as proliferation, migration/alignment, and apoptosis, we further examined each functional aspect individually. To evaluate cell viability, HUVEC were treated with increasing concentrations of drug or isotype control for 72 h, followed by the addition of the MTS tetrazolium compound, which undergoes a color change when bioreduced by metabolically active cells. While little to no effect of IgG was observed at the ng/ml range (data not shown), IgG at the μg/ml range inhibited HUVEC viability. At this dose range, TRC105 (μg/ml) elicited comparable inhibition to IgG (μg/ml) alone, suggesting the inhibition observed in response to TRC105 was an IgG non-specific effect. In regular medium, bevacizumab showed no appreciable inhibition on HUVEC viability when tested at concentrations of 100 ng/ml to 1 μg/ml (Fig. 2a). Higher concentrations of bevacizumab (100 μg/ml to 1 mg/ml) were also tested, but again, the effects were negligible (data not shown). When both drugs were combined, the inhibitory effect on HUVEC viability was essentially the same as IgG alone.

The lack of a bevacizumab effect on HUVEC viability in serum-containing medium was not surprising. The high abundance of growth factors present in the serum-containing medium, including FGF, EGF, VEGF, and IGF, all can serve as potential compensatory factors upon VEGF blockage. To isolate the effect of TRC105 and bevacizumab on VEGF-specific cell viability, we optimized a nutrient-limited medium that excluded all other growth factors (i.e., FGF, EGF, and IGF) except VEGF. Limited medium was formulated containing 0.5 % FBS and 30 ng/ml VEGF. In this setting, bevacizumab produced a significant drug-specific dose-dependent inhibition on HUVEC viability (*p* < 0.01 vs. IgG control, Fig. 2b), while TRC105-mediated inhibition at μg/ml concentrations was less robust and non-specific. A closer comparison among bevacizumab alone, bevacizumab + IgG, bevacizumab + TRC105 treated cells revealed little enhancement when TRC105 was added to bevacizumab. Therefore, the combination of both drugs did not lead to greater inhibition of HUVEC viability as compared to bevacizumab alone.

### TRC105 and bevacizumab inhibit HUVEC migration

**Fig. 3:**
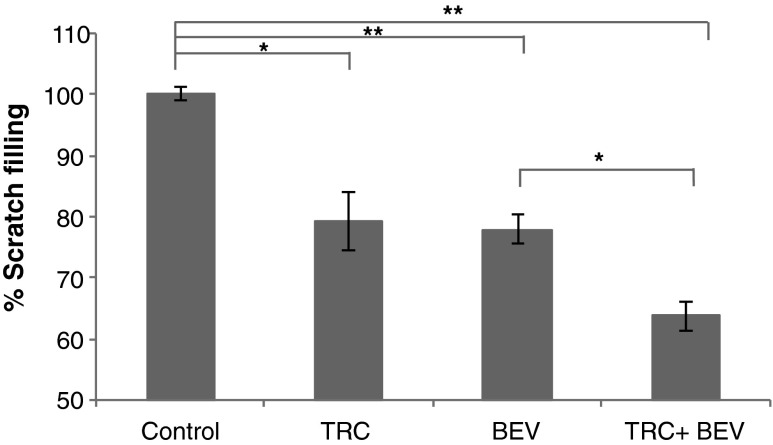
TRC105 and bevacizumab inhibit HUVEC migration. HUVEC confluent monolayers were scratched to create a gap, exposed to TRC105 (100 μg/ml), bevacizumab (100 ng/ml), or the combination of TRC105 (100 μg/ml) and bevacizumab (100 ng/ml), and monitored for cell migration for 16 h. Percentage of scratch filling at 10 h post scratch was shown. Each column represents the mean ± SD of three independent experiments. * *p* < 0.05; ** *p* < 0.01 compared to no drug control

The in vitro scratch assay was used to measure cell motility [17]. HUVEC cells were allowed to grow to confluence, then a gap was introduced into the cell monolayer and the process of cell migration was monitored over a period of 16 h. In regular medium, untreated cells refilled the gap within 10–11 h. As shown in Fig. 3, incubation of HUVEC in the presence of 100 μg/ml TRC105 significantly delayed endothelial cell migration (*p* < 0.05). Treatment with 100 ng/ml bevacizumab achieved similar effects (*p* < 0.01). Treatment with both drugs inhibited migration more than observed with either TRC105 or bevacizumab alone (*p* ≤ 0.01) (Fig. 3). IgG isotype control at 100 μg/ml showed no inhibition on HUVEC migration (data not shown). When scratch filling was assessed in limited medium, HUVEC cells did not migrate well and never reached confluence in the absence of drugs, even after 24 h post scratch.

### TRC105 and bevacizumab induce HUVEC apoptosis

**Fig. 4:**
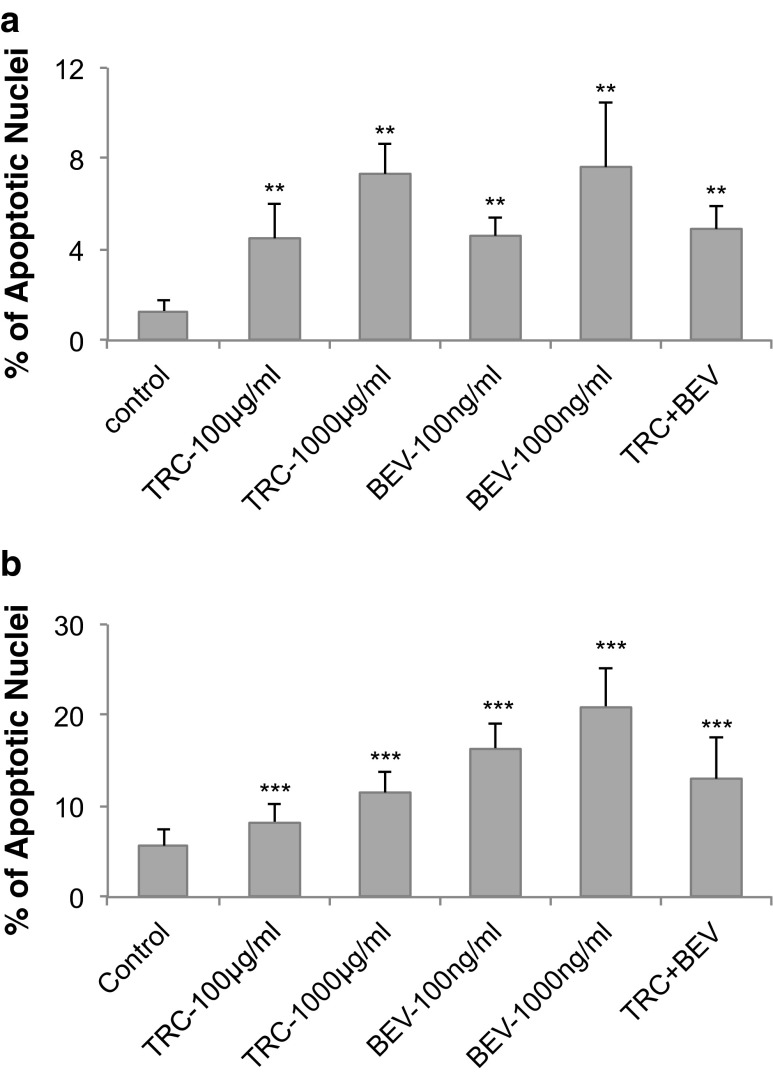
TRC105 and bevacizumab induce HUVEC apoptosis. Apoptosis was evaluated by Hoechst staining after HUVEC were treated with TRC105 (100 or 1,000 μg/ml), bevacizumab (100 or 1,000 ng/ml), or the combination of drugs at the lower doses (100 μg/ml TRC105 and 100 ng/ml bevacizumab) for 3 days. **a**. Regular medium. **b**. Limited medium. Each column represents the mean ± SD of three independent experiments. ** *p* < 0.01, *** *p* < 0.001 compared to no drug control

To test the apoptotic effects of TRC105 and bevacizumab, HUVEC were first treated with each drug individually in regular or limited medium for 72 h, and then subjected to Hoechst staining (Fig. 4). Condensed, fragmented nuclei representing apoptotic features were counted, and the ratio of apoptotic nuclei vs. total nuclei was plotted. Camptothecin (4 μg/ml) was used as a positive control and induced more than a 30-fold increase in the apoptotic cell ratio after overnight incubation (data not shown). IgG isotype control was indistinguishable from the no drug control (data not shown) [18]. In regular medium, TRC105 exhibited a small, yet significant, dose-dependent induction of apoptosis, with a 3-fold increase at 100 μg/ml, and a 6-fold increase at 1,000 μg/ml, versus no-drug control (Fig. 4a). Bevacizumab induced apoptosis to similar levels as seen with TRC105 (*p* < 0.01 vs. control). Additive effects were not observed when the drugs were combined, as the induction of apoptosis was similar compared to either single agent.

In limited medium, the basal apoptosis rate was higher than that in regular medium (5 % vs. 1.2 %). TRC105 elicited a 2-fold induction of apoptosis at 1,000 μg/ml. Bevacizumab was a more potent inducer of apoptosis under these conditions, achieving a 3.8 fold increase at 1,000 ng/ml (*p* < 0.001 vs. control). As was seen with regular medium, the combination of drugs in limited medium did not further increase apoptosis (Fig. 4b).

### TRC105 differentially modulates Smad 1/5/8 and Smad 2/3 signaling pathways in response to BMP-9 and TGF-β1

**Fig. 5:**
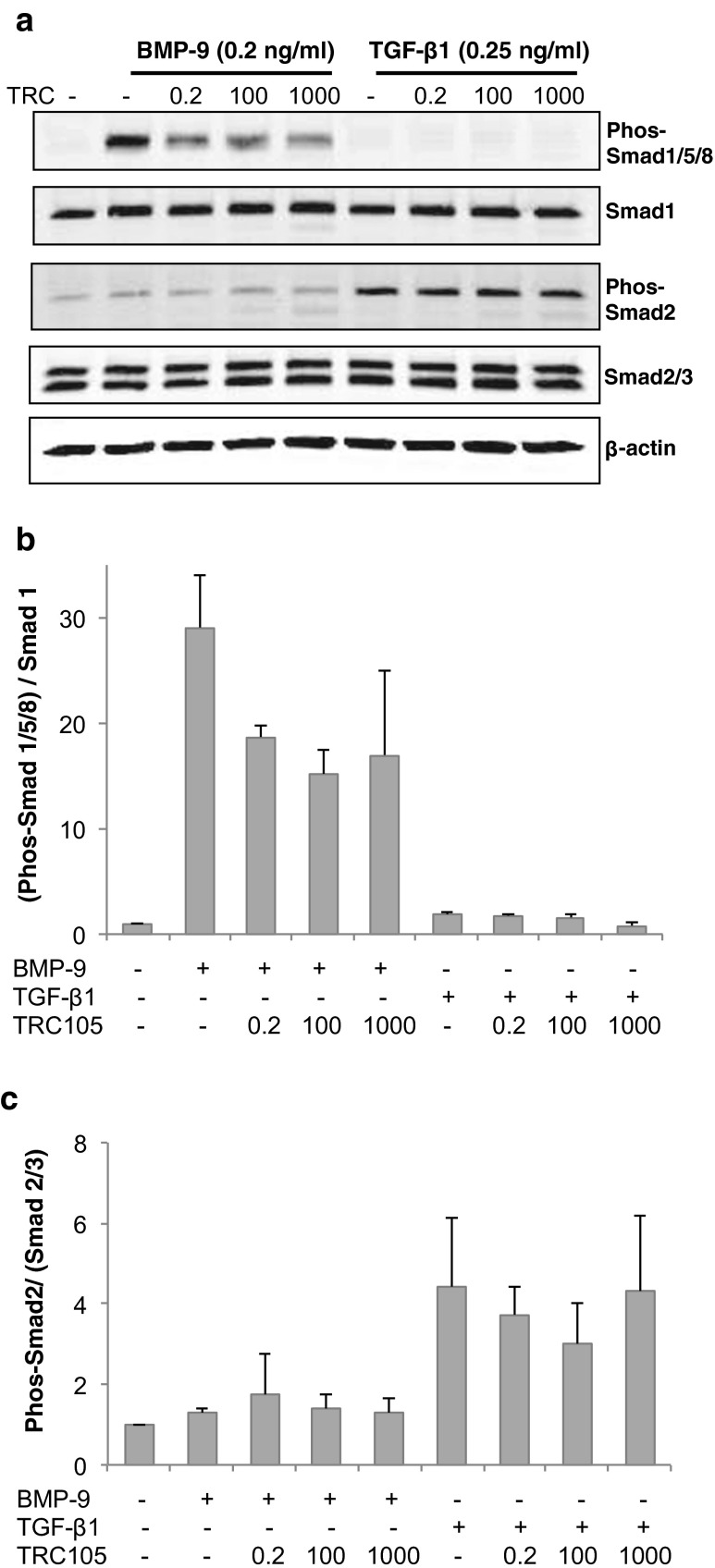
TRC105 diminished BMP-9 induced Smad 1/5/8 signaling in HUVEC cells. **a**. Western blotting showing the levels of phosphorylated Smad 1/5/8, total Smad 1, phos-Smad 2, total Smad 2/3 in response to the stimulation of BMP-9 or TGF-β1. Cells were pre-incubated with various doses of TRC105 for 1 h prior to stimulation. **b**. Effect on Smad 1/5/8 signaling as revealed by the ratio of phos-Smad 1/5/8 vs. total Smad 1. **c**. Effect on Smad 2 signaling as revealed by the ratio of phos-Smad 2 vs. total Smad 2/3. 0.2, 100, 1,000 depict TRC105 doses of 0.2, 100, and 1,000 μg/ml, respectively

Multiple factors have been identified as endoglin ligands, including TGF-β1 and TGF-β3 [7], as well as BMP-9 and BMP-10 [19]. To investigate these signaling modalities, serum-starved HUVEC cells were exposed to either BMP-9 or TGF-β1. As shown in Fig. 5, BMP-9 strongly activated Smad 1/5/8 phosphorylation (~30 fold), but had little effect on Smad 2. In contrast, TGF-β1 exposure mainly activated Smad 2 phosphorylation (~5 fold), but not Smad 1/5/8 signaling.

To explore the underlying molecular mechanisms contributing to the effects of TRC105 on HUVEC function, the phosphorylation status of Smad 1/5/8 and Smad 2 was investigated. When HUVEC were pre-incubated with increasing amounts of TRC105, BMP-9-induced Smad 1/5/8 phosphorylation was inhibited across all doses of TRC105 tested (Fig. 5, panel b), including a dose of 0.2 μg/mL, which is the TRC105 concentration expected, based on binding avidity studies, to saturate endoglin binding sites on HUVECs. In contrast, TRC105 treatment only marginally modulated TGF-β1 induced Smad 2 phosphorylation (panel C). Both isotype control IgG and bevacizumab exhibited negligible inhibition of either Smad 1/5/8 or Smad 2 phosphorylation (data not shown).

## Discussion

In this study, we evaluated the anti-angiogenic drug TRC105, and its combination with bevacizumab, in a series of HUVEC functional assays. Initially, individual drugs were tested over a broad range of dosing levels under each assay condition. In every assay system, doses that induced moderate effects (typically around 25–30 % inhibition) were chosen for these analyses in order to detect potential additive or synergistic effects with both drugs. For most assays, the dose of TRC105 was empirically determined to be 100 μg/ml, as lower doses exhibited little effect on HUVEC function. This dose is clinically relevant as pharmacokinetic analyses revealed that the serum concentration of TRC105 following dosing in advanced cancer patients at the recommended phase 2 dose ranged from 200 to 600 μg/ml [16]. For bevacizumab, the dose tested in most assays was empirically determined to be 100 ng/ml.

Despite the fact that TRC105 and bevacizumab are both monoclonal antibodies that block angiogenesis, the targets of each drug are different and utilize distinct mechanisms. Endoglin is an integral cell membrane receptor located on proliferating endothelial cells [20], whereas VEGF is a soluble angiogenic factor that is primarily released by tumor cells and tumor-associated stromal cells [21, 22]. Therefore, combining TRC105 and bevacizumab has the potential to block complementary pathways leading to improved efficacy.

We observed that individually, both TRC105 and bevacizumab blocked HUVEC tube formation, and the most robust inhibition was achieved when both drugs were combined (Fig. 1). The tube formation assay is a powerful tool to monitor ECs during vascular network formation, representing an end-point evaluation of the complicated interplay among many processes, including proliferation, differentiation, migration, apoptosis, etc. To further interrogate these processes, we next investigated specific EC functional biology in more defined assay systems.

When given at 100–1,000 μg/ml levels, TRC105 and its isotype control (IgG) similarly inhibited HUVEC viability, in both regular and nutrient-limited medium (Fig. 2). In contrast, although bevacizumab elicited little effect on HUVEC viability in regular medium, it showed dose-dependent, drug-specific inhibition in limited medium (Fig. 2b). While TRC105 had little effect on HUVEC viability, several observations are noteworthy. First, contrary to bevacizumab, TRC105 elicited no inhibitory effect on HUVEC growth in nutrient-limited medium. Second, given that the steady state plasma concentration of TRC105 in patients is in μg/ml range, the potential non-specific effect of IgG on EC growth should be considered. Third, Anderberg et al., reported that genetic knockdown of endoglin sensitizes tumors to VEGF inhibition [23], advocating for the potential benefit of co-administration of these drugs. The underlying mechanism is unlikely to be an effect on EC growth/viability, as suggested by our MTS data and suggests that the inhibition of HUVEC tube formation is not due to the impact of these agents on cell viability.

The effect of endoglin on cell motility is controversial. While endoglin is considered to be an inhibitor of cell migration through its extracellular RGD domain that binds to intercellular matrix proteins [24], evidence exists that endoglin promotes ALK1 signaling, leading to increased cell mobility [25]. Additionally, endoglin has been shown to antagonize the inhibitory effect of TGF-β1 on HUVEC migration, suggesting a positive role of endoglin in HUVEC motility [26]. Our scratch assay data support the latter hypothesis (Fig. 3). TRC105 moderately decreased HUVEC migration, as quantified by a reduced percentage of cells that migrated into the gap created by the scratch. Bevacizumab exhibited similar inhibitory effects, consistent with VEGF’s motility-promoting role [27]. The combination of both drugs exhibited an additive inhibition when compared to either drug alone in the scratch filling assay.

EC apoptosis provided another opportunity to evaluate TRC105 and bevacizumab in both regular and limited medium. In the presence of multiple growth factors, TRC105 and bevacizumab exhibited similar potency in inducing HUVEC apoptosis (Fig. 4a). In limited medium, where VEGF is essentially the only growth factor, TRC105 was also active and exhibited dose-dependent induction of HUVEC apoptosis (Fig. 4b). These findings are in agreement with previous observations that SN6j, the parent antibody of TRC105, also led to increased HUVEC apoptosis in vitro [18]. However, there were no additive effects by combining both drugs.

Lastly, we explored the molecular mechanisms underlying the ability of TRC105 to block HUVEC function. Since endoglin is a type III receptor for TGF-β and BMP, we investigated the effects of TRC105 on Smad signaling, pathways known to play pivotal roles in EC proliferation and viability. In assessing Smad phosphorylation (Fig. 5), three doses of TRC105 were chosen: 0.2, 100, and 1,000 μg/ml. A dose of 0.2 μg/ml is the target concentration predicted to saturate endoglin on cell surface [16]. Doses of 100 and 1,000 μg/ml fall within or close to the range of TRC105 plasma concentrations (200–600 μg/ml) achieved in cancer patients following dosing at the recommended phase 2 dose [16].

While endoglin’s role in canonical TGF-β signaling is well documented [28], recent data indicate that BMP-9 and −10 are the important endoglin ligands that mediate Smad 1/5/8 phosphorylation needed for activation of primary EC [10, 29]. Our data corroborate that BMP-9 effectively stimulates Smad 1/5/8 phosphorylation (more than 30 fold induction), while TGF-β1 only modestly stimulates Smad 2/3 phosphorylation (approximately 5 fold induction). Our observation is consistent with the model suggesting that the BMP9-ALK1-Smad1/5/8 and the TGFβ-ALK5-Smad2/3 axes co-exist in parallel in primary human EC [10].

When HUVEC were pre-treated with TRC105, BMP-9-induced Smad 1/5/8 phosphorylation was inhibited by 50 %, even at the lowest dose of 0.2 μg/ml (Fig. 5b). In contrast, TGF-β1-induced Smad 2/3 phosphorylation was less affected (<20 % inhibition). It is well established that Smad 1/5/8 signaling promotes EC activation. Therefore, the inhibition of this pathway exerted by TRC105 would lead to EC deactivation, contributing to the anti-angiogenic, anti-tumor function of TRC105.

In addition to blocking BMP9/Smad1/5/8 signaling, other mechanisms may be responsible for TRC105 anti-angiogenic function. For example, biomarker analyses from cancer patients treated with escalating doses of TRC105 revealed significant increases of soluble endoglin (sEnd) after TRC105 administration [30]. Kumar et al. proposed two mechanisms as to how TRC105 could induce sEnd shedding [31]. First, TRC105 could stabilize endoglin/MMP-14 complexes on the cell surface; second, TRC105 could induce MMP-14 gene expression to facilitate enzymatic cleavage of endoglin. In either event, sEnd would be released and serve as a trap for its ligand BMP-9, further diminishing the BMP9/Smad 1/5/8 pathway [32]. Another mechanism may involve the intracellular domain of endoglin. This domain contains important structural elements that serve as docking sites for multiple adaptor proteins, such as zyxin, zyxin-related protein 1 [33], β-arrestin2 [34], and GIPC [35]. It remains unclear how TRC105 binding to endoglin would affect these proteins and what role (s) the intracellular domain of endoglin plays in TRC105 function.

Clinically relevant doses of TRC105 were tested in these in vitro cell-based assays and could facilitate our understanding of the efficacy in vivo. Based on the superior effects of TRC105 and bevacizumab in HUVEC tube formation assays, the combination of both drugs has the potential to increase drug efficacy and reduce resistance. Currently, developing rationale-based combinations of multiple anti-angiogenic agents is a favored strategy to overcome resistance in the clinic [36]. Bevacizumab has been successfully added to chemo- and radiotherapy regimens and has significantly improved patient outcomes [37]. In the case of TRC105 and bevacizumab, with each drug targeting an independent pathway, more effective blockage of angiogenesis is expected. However, caution needs to be taken when determining drug dosing, relative ratio, frequency of administration, etc., to prevent the possible convergence of downstream effectors becoming exhausted or saturated.

In summary, we have demonstrated that the combination of TRC105 and bevacizumab led to greater inhibition in HUVEC functional assays, such as tube formation and migration, than either drug alone. We also explored Smad signaling and confirmed that diminished BMP9/Smad 1/5/8 signaling is a mechanism contributing to TRC105’s anti-angiogenic, anti-tumor effect. The superior potency demonstrated by the drug combination advocates their co-administration in vivo as a therapeutic strategy.
